# Selective hippocampal transcriptional adaptation to long-term cannabidiol exposure in mice

**DOI:** 10.1186/s12864-026-13028-8

**Published:** 2026-06-08

**Authors:** J Żurowski, E Ocłoń, I Jasielczuk, T Szmatoła, K Mizera-Szpilka, T Taubner, L Szumiec, E Semik-Gurgul, A Gurgul

**Affiliations:** 1 Faculty of Veterinary Medicine, Department of Basic Sciences, University of Agriculture in Kraków, Redzina 1C, Krakow, 30-248 Poland; 2 Faculty of Veterinary Medicine, Laboratory of Recombinant Proteins, University of Agriculture in Kraków, Redzina 1C, Krakow, 30-248 Poland; 3 Faculty of Veterinary Medicine, Department of Infectious Disease and Public Health Protection, University of Agriculture in Kraków, Redzina 1C, Krakow, 30-248 Poland; 4 Department of Nutritional Physiology and Animal Product Quality, Institute of Animal Science, Přátelství 815, Prague, 104 00 Czech Republic; 5 Department of Molecular Neuropharmacology, Maj Institute of Pharmacology of the Polish Academy of Sciences, Smętna 12, Kraków, 31-343 Poland; 6 Department of Animal Molecular Biology, National Research Institute of Animal Production, Krakowska 1, Balice, 32-083 Poland

**Keywords:** Cannabidiol, Hippocampus, Mitochondrial bioenergetics, RNA-seq

## Abstract

**Background:**

CBD is widely studied for its stress-reduction and cognitive-enhancing properties, but its effects on hippocampal molecular organisation under physiological settings are unknown. Acute and long-term intraperitoneal CBD treatment at different doses was tested on hippocampus gene expression, circulating corticosterone, and behavioural performance in C57BL/6J mice.

**Results:**

Short-term administration did not induce detectable transcriptional changes. In contrast, long-term treatment with 10 mg/kg CBD, but not lower or higher doses, resulted in significant hippocampal transcriptional remodelling. Overrepresentation analysis showed coordinated control of mitochondrial oxidative phosphorylation genes, particularly numerous respiratory chain complex I components, and purine and nucleotide metabolic pathways. Shared mitochondrial respiratory genes, not classical disease-associated effectors, enriched KEGG categories for neurodegeneration and retrograde endocannabinoid signalling. The endocrine profile showed a temporary increase in circulating corticosterone after short-term exposure, but long-term dosing decreased it. Behavioural effects were modest and limited across paradigms.

**Conclusions:**

These results show that long-term administration of an intermediate CBD dose alters subsets of genes related to coordinated bioenergetic and nucleotide-related transcriptional adaptation in the hippocampus, which modulates endocrine stress markers but does not disrupt behaviour. The data suggest that chronic CBD exposure may cause metabolic recalibration in stress-sensitive brain circuits rather than acute neuromolecular reprogramming.

**Supplementary Information:**

The online version contains supplementary material available at 10.1186/s12864-026-13028-8.

## Introduction

Cannabidiol (CBD), a major non-intoxicating phytocannabinoid extracted from *Cannabis sativa*, has attracted significant clinical and scientific attention. In the United States, pure CBD is sanctioned by the U.S. Food and Drug Administration as an antiseizure treatment and is sold under the brand name Epidiolex [1]. In addition to its anticonvulsant properties, CBD has been documented to possess anti-inflammatory, antioxidant, anxiolytic, antipsychotic, neuroprotective, and analgesic activities [2]. Despite thorough investigation, the molecular mechanisms underlying these varied biological activities remain only partially elucidated and likely to require intricate and partially overlapping signaling pathways.

An extensive body of research demonstrates that CBD has notable effects on the central nervous system, especially in the hippocampus [3–6]. The hippocampus is fundamentally engaged in spatial navigation by creating cognitive maps facilitated by place cell activity, and is vital for memory consolidation [7]. Alongside the amygdala, it plays a role in the regulation of stress and anxiety-related processes [8, 9], areas often noted to be influenced by CBD.

Experimental investigations indicate that CBD affects various physiological parameters in the hippocampus. It mitigates hippocampus hyperexcitability, partially by antagonizing the pro-excitatory actions of lysophosphatidylinositol at GPR55 receptors [10]. In neurodegenerative models, CBD improves neuronal survival and decreases oxidative stress, alleviating amyloid-β-induced neurotoxicity in hippocampal neurons [11]. Transcriptomic analyses reveal that CBD administration alters hippocampal gene expression, resulting in the downregulation of genes related to mitochondrial electron transport and ribosome biogenesis, as well as the upregulation of genes involved in chromatin remodelling and synaptic organization in the CA1 region [12]. These findings indicate that CBD may affect cellular metabolism, protein synthesis, and synapse structure, therefore leading to changes in neuroplasticity. Furthermore, CBD has been reported to change hippocampal ripple dynamics during sleep, potentially influencing memory consolidation processes [13].

This study aimed to examine the effects of acute and long-term cannabidiol administration on hippocampus transcriptional remodeling, circulating corticosterone levels, and behavioral performance in C57BL/6J mice under physiological conditions. Corticosterone levels were measured as an indicator of hypothalamic–pituitary–adrenal axis activity, while behavioral paradigms were employed to test anxiety-related reactions and spatial memory. In parallel, hippocampal transcriptome profiling was performed to establish molecular changes associated with CBD exposure.

## Materials and methods

### Animals

48 male mice of the standard C57BL/6J strain and an initial age of 60 days (purchased from the Department of Clinical Immunology and Transplantology of the Jagiellonian University) were used in the experiment. The animals were kept in a designated building, in groups of 6 in conventional polycarbonate cages measuring 37 × 21 × 15 cm, following the requirements specified in the Regulation of the Ministry of Agriculture and Rural Development (Poland, under EU regulations). The cages were placed on racks with rails and equipped with water bottles and food troughs. An air conditioner provided ventilation and heating of the rooms with 15 air changes per hour. The mice were kept at a temperature of 22 ± 2 °C and humidity of 55 ± 10%, in a light cycle of 12:12 h. All animals had constant access to water and standard chow. Environmental enrichment was provided by using appropriate bedding and additional cage equipment (wooden blocks and a system of tubes). Animals were monitored daily in the short-term group (I) and every third day in the long-term group (II) to assess general health and well-being.

### CBD treatment and tissue sampling

All animal procedures were evaluated and accepted by an II Local Ethics Committee in Kraków (permission number 90/2022).

**Fig. 1:**
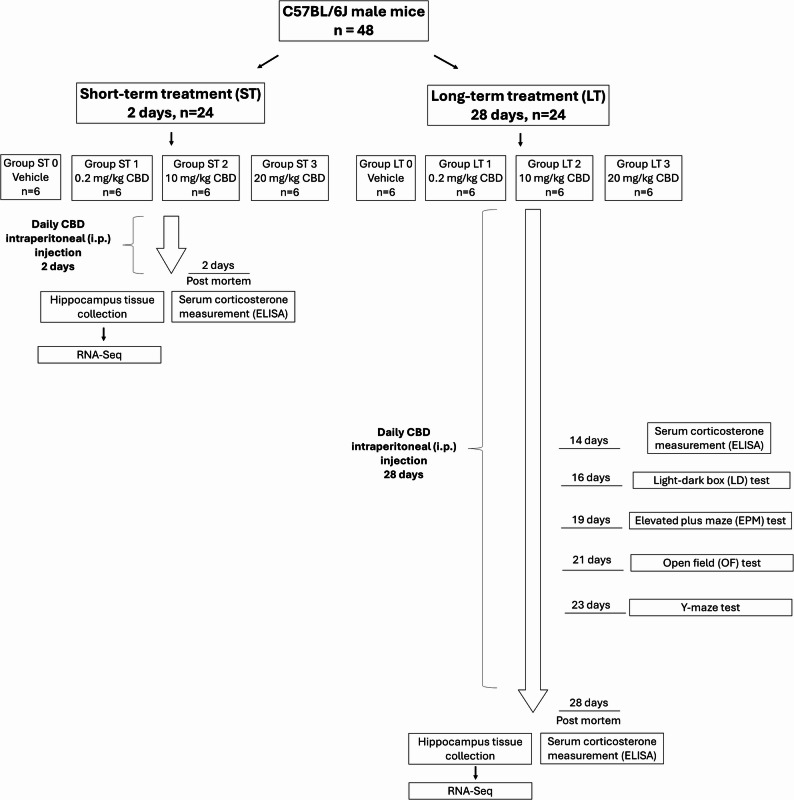
Schematic representation of the experimental design

The animals were randomly classified into two major groups: (ST) short-term CBD administration (accidental use; 2 days; *n* = 24) and (LT) long-term CBD administration (long-term, 28 days; *n* = 24). In each of the main groups, four subgroups including six mice each received different doses of CBD, ranging from a low dose commonly used in supplementation (0.2 mg/kg bw) [14], through a medium (10 mg/kg bw) [14], to a medium-high dose applied in selected clinical trials (20 mg/kg bw) [15]. Additionally, a control group (placebo/vehicle) was maintained in each main group. Schematic experiment setup can be found in Fig. 1. Cannabidiol with a purity exceeding 99% (NMID512B, MERCK, USA) was dissolved in sterile saline containing 2% Tween 80 (v/v) and administered once daily in a 200 µL via intraperitoneal (i.p.) injection to ensure systemic bioavailability. Stock solutions were prepared every two days at concentrations calculated based on the mean body weight of each experimental group, allowing the intended dose to be delivered in a fixed injection volume of 200 µL per animal. Animals were weighed weekly to update the mean group body weight and recalculate the required concentration accordingly. As a vehicle, 200 µL saline, containing 2% Tween 80, was used.

Following the final CBD administration, animals were euthanized at least 35 min after the last injection, following a 12 h fasting period. Euthanasia was performed under isoflurane anesthesia followed by cervical dislocation. Brains were rapidly removed, and the entire left and right hippocampi were carefully dissected on ice. Tissue samples were immediately transferred into 1.5 mL tubes containing 200 µL of stayRNA™ reagent (A&A Biotechnology, Poland) to stabilize RNA and prevent degradation. After overnight incubation in stayRNA™ according to the manufacturer’s protocol, the reagent was removed, and samples were stored at − 20 °C until RNA isolation.

### Blood sampling and assessment of plasma corticosterone levels

Blood samples were collected at four predefined time points: before CBD administration as baseline, after short-term treatment, on day 14 of long-term treatment, and at the end of long-term treatment (Fig. 1). Approximately 60 µL of blood was obtained from the lateral tail vein into tubes containing K3-EDTA. When the collected volume was insufficient for analysis, plasma from two animals within the same experimental group was pooled to obtain the required volume while maintaining a minimum of three biological replicates (each obtained from two different animals) per group. Whole blood was centrifuged at 2,000 × g for 10 min at 4 °C. Plasma was separated and stored at − 20 °C until analysis.

Plasma corticosterone levels were measured with a competitive ELISA kit (EIACORT) from Thermo Fisher Scientific (USA), following the manufacturer’s guidelines. Absorbance was quantified at 450 nm with a TECAN (Switzerland) Infinite M200 PRO microplate reader. The intra-assay and inter-assay coefficients of variation were 5.2% and 7.9%, respectively.

### Behavioral tests

In the middle of the experiment (after 16–23 days of CBD administration in the LT group, *n* = 24), behavioral tests (Y-maze, light-dark box, Open field, and Elevated plus maze) were conducted to assess spatial memory, cognitive abilities, and the stress levels in the CBD-treated mice and controls. All tests were taken in a quiet, darkened room with controlled light intensity and temperature (21 °C). Before tests, mice were injected with CBD and adapted to the new room during a 30–40 min stay in cages. After that, they were tested alternately from different groups in a single day. The Light-dark box (LD) test, which evaluated anxiety, was performed on day 16 of the experiment. The bright part of the box was illuminated with an intensity of around 400 lx, while the dark part had less than 50 lx. During a 300-second test duration, parameters such as the percentage of time spent in the bright part, latency to enter the bright part, risk assessments, and the number of transitions between box parts were assessed manually [16]. Another anxiety-related Elevated plus maze (EPM) test was performed on day 19 of the experiment. In this test, through 300 s of test duration, parameters such as latency and % of time spent in open arms, risk assessments and number of entries to the open arms were assessed, as described in Komada et al. (2008) [17]. The open field (OF) test, also for anxiety level, was performed on day 21 of the CBD administration. During this test, which was run for 600 s, parameters such as the percentage of time spent in the central part of the box (10 × 10 cm size, 200 lx), latency, and total number of passes to the central part were manually determined. The test was performed as previously described [18]. The Y-maze test was performed on day 23 of CBD administration. In this test, throughout 300 s of the test duration, parameters such as: spontaneous alteration performance (SAP; indicates good functioning of working memory), same arm returns (SAR) and alternate arm returns (AAR), were determined (as described in [19]). In behavioural experiments, single animals were excluded if they showed no exploratory activity in the apparatus (e.g., no entries into maze arms or zones), precluding reliable behavioral assessment. Additionally, some data points were excluded only in cases of clearly identifiable technical errors during manual test execution.

### RNA purification and RNA-Seq library construction

After tissue defrosting on ice, RNA was purified using AllPrep DNA/RNA Mini Kit (Qiagen, Germany) following the standard protocol. The recovered RNA was quality assessed using the TapeStation 4150 system (Agilent Technologies, USA) and quantified using Qubit system (Thermo Fisher Scientific, USA). 200 ng of the purified RNA (high-quality RNA; RIN > 7) was used to prepare directional libraries for high-throughput transcriptome sequencing, using CORALL mRNA-Seq V2 (Lexogen, Austria) kit. The libraries were sequenced commercially in a 2 × 150 bp run on NovaSeq6000 Illumina (USA) system. Raw sequencing reads were deposited in the Gene Expression Omnibus (GEO) NCBI database, under accession number GSE276463 and Sort Read Archive (SRA) database with BioProject number PRJNA1157422.

### RNA-Seq data analysis

The raw sequence reads underwent quality control using FastQC (v0.11.9). Following this, Flexbar (3.5.0) [20] was used to trim and filter the sequences, removing low-quality bases, adapter sequences, and reads that were too short after trimming. The filtered reads were then aligned to the mouse reference genome GRCm39 using the STAR aligner (2.7.5c) [21]. The aligned reads were counted with Htseq-count (1.99.2) [22]. Normalisation of the read counts and differential expression analysis using DESeq2 (Wald test within a negative binomial generalised linear model) [23], were performed within the iDEP2.0 platform (integrated Differential Expression & Pathway analysis, v2.01) [24].

iDEP2.0 was also utilized to analyze expression profile variability through principal component analysis (PCA) and hierarchical clustering based on Euclidean distance. Additionally, Gene Set Enrichment Analysis for differentially expressed genes in biological processes (BP) from the Gene Ontology (GO) database [25] and Kyoto Encyclopedia of Genes and Genomes (KEGG) terms were carried out using WEB-based GEne SeT AnaLysis Toolkit (WebGestalt) [26].

Genes and processes were considered significant if the adjusted p-value, corrected for multiple testing using the Benjamini-Hochberg method (FDR) [27], was < 0.1.

### qPCR validation

For RNA-Seq validation using quantitative real-time polymerase chain reaction (RT-qPCR), five differentially expressed genes that were common to at least two RNA-Seq comparisons were selected. The analysed genes included both upregulated (*Wdr33*,* Ptpru*) and downregulated (*Snapc5*,* Tmem258*,* Emcn*) transcripts. Validation was performed on samples from LT treatment groups in which differential expression was detected, i.e. animals receiving 10 and 20 mg/kg body weight (b.w.) of CBD.

For RT-qPCR analysis, cDNA was synthesised from 300 ng of total RNA using the High-Capacity cDNA Reverse Transcription Kit (Thermo Fisher Scientific) according to the manufacturer’s instructions. RT-qPCR was carried out using the AmpliQ 5× HOT EvaGreen^®^ qPCR Mix Plus (ROX) (Novazym, Poznań, Poland) and primers designed to span two adjacent exons of the target mRNA sequences (Supplementary File 1). Each sample was analysed in triplicate on a QuantStudio™ 7 Flex Real-Time PCR System (Thermo Fisher Scientific). Relative gene expression levels were calculated using the ΔΔCt method, with *Pgk1* and *Rps18* used as reference genes for normalization [28].

### Statistical analysis

Hormone levels and behavioural test parameters were analysed using JASP statistical software. After initial evaluation of data distribution using the Shapiro-Wilk test, ANOVA or Kruskal-Wallis ANOVA were used for normally and not-normally distributed data, respectively. Subsequently, post-hoc Tukey (normal distribution) or Dunn (nonparametric) tests were used for these data types to resolve pairwise comparisons. The consistency between RNA-Seq and RT-qPCR results was assessed by analysing correlation coefficients between the two methods using JASP statistical software.

## Results

### Changes in plasma corticosterone levels following CBD treatment

Plasma corticosterone concentrations were determined after 14 days in the long-term (LT) group and post-mortem after 2 days (short-term, ST group) and 28 days (LT group) of CBD administration. After 2 days of ST treatment, corticosterone levels were significantly elevated in animals receiving 0.2 mg/kg b.w. (16.7 ± 0.9 ng/µl, *p* < 0.01) and 20 mg/kg b.w. (16.2 ± 1.2 ng/µl, *p* < 0.01) compared with controls (13.6 ± 1.1 ng/µl). The intermediate dose (10 mg/kg b.w.; 14.5 ± 1.0 ng/µl) revealed no significant difference compared to the control group.

By day 14 of LT treatment, a dose-dependent decrease in corticosterone concentrations was observed. The administration of 10 mg/kg b.w. resulted in a reduction of hormone levels by roughly 25% (8.0 ± 0.9 ng/µl, *p* < 0.05), whereas 20 mg/kg body weight elicited a more significant decrease of nearly 45% (6.1 ± 0.7 ng/µl, *p* < 0.001). The lowest dose (0.2 mg/kg b.w.; 9.8 ± 1.0 ng/µl) did not differ significantly from controls (10.6 ± 0.8 ng/µl).

**Fig. 2:**
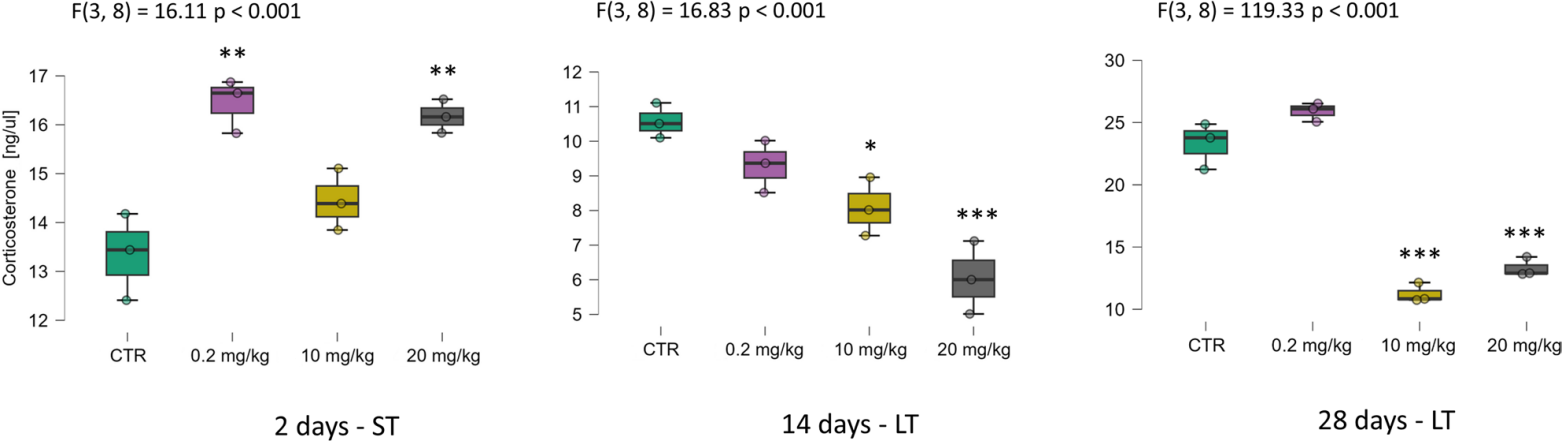
Changes in serum corticosterone concentration following CBD treatment, as measured by ELISA. *p* < 0.05*, *p* < 0.01**P < 0.001***; statistical significance is marked only for comparisons against the control group. *p* – *p*-value of global ANOVA tests. F(df1, df2) – ANOVA test statistics with the presented degrees of freedom; CTR – control; ST – short-term CBD treatment; LT – long-term CBD treatment

After 28 days of treatment, corticosterone levels in the 0.2 mg/kg b.w. group (26.1 ± 2.4 ng/µl) were similar to those of the control group (23.7 ± 1.9 ng/µl). In contrast, the 10 mg/kg b.w. dose diminished corticosterone levels by roughly 55% (10.7 ± 1.1 ng/µl, *p* < 0.001), whereas the 20 mg/kg b.w. dose led to an estimated 40% reduction (14.1 ± 1.3 ng/µl, *p* < 0.001) (Fig. 2).

### Assessment of behaviour alterations in CBD-treated mice

Behavioural alterations following CBD treatment were assessed using the Light–Dark box, the Y-maze, Open Field and Elevated plus Maze Performance tests.

**Fig. 3:**
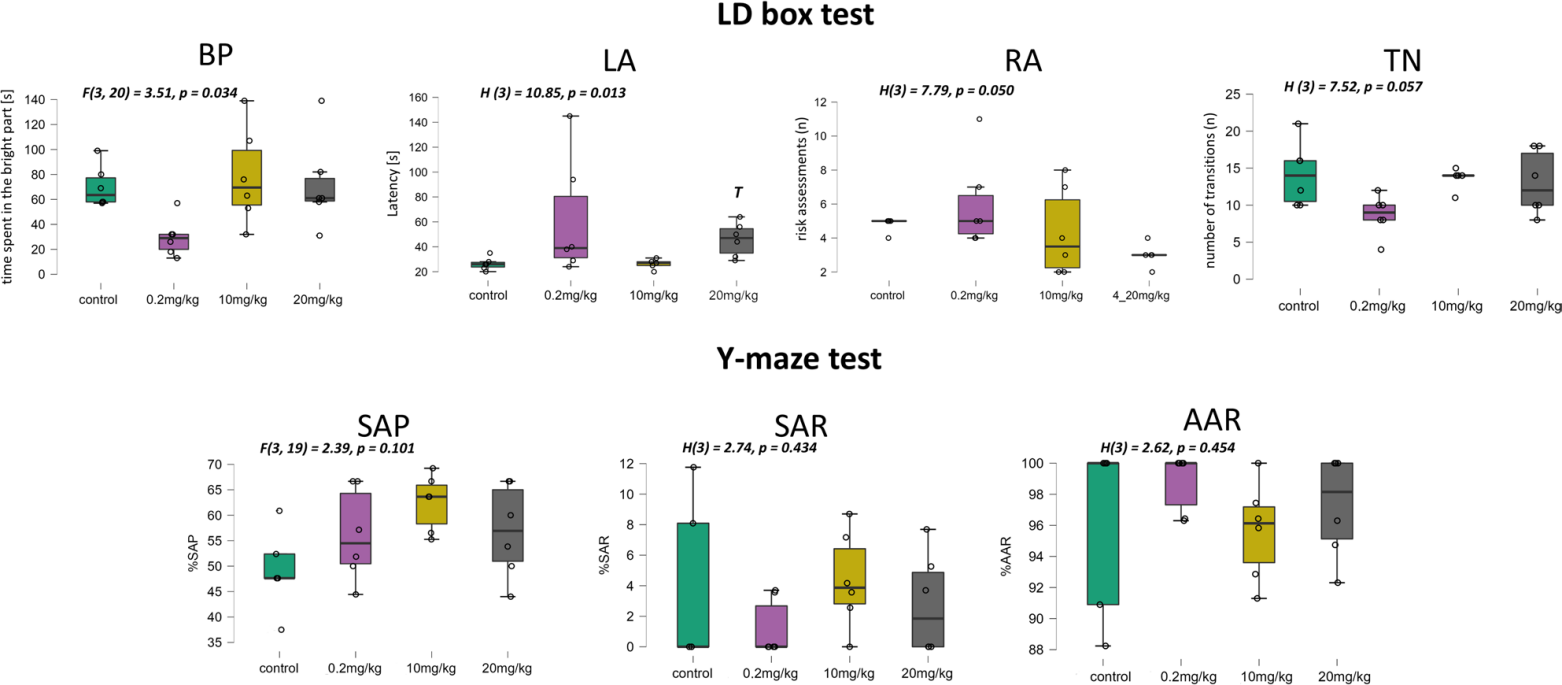
Results of light-dark (LD) and Y-maze behavioural tests. BP - percentage of time spent in the bright part; LA - latency to enter the bright part; RA - risk assessments; TN - the number of transitions between box parts; SAP - spontaneous alteration performance; SAR - same arm returns; AAR - alternate arm returns; *p* – *p*-value of ANOVA (F) or Kruskal-Wallis (H) test; F(df1, df2) – ANOVA test statistics with the presented degrees of freedom; H(df1) - Kruskal-Wallis test statistics with the presented degrees of freedom; T – statistical trend against control group

In the Light–Dark box test, CBD administration produced effects on anxiety-related behaviours (time spent in the bright part) as shown by the global ANOVA test (*p* < 0.05), which, however, were not confirmed in post-hoc tests. Similar effects were observed for latency, risk assessments and number of transitions among light-dark parts, which showed a clear statistical trend without significance at pairwise comparisons (Fig. 3).

In the Y-maze test, no significant differences (*p* > 0.05) were detected in spontaneous alternation performance (%SAP) or alternate arm return (%AAR) across treatment groups. These findings suggest that CBD did not disrupt short-term memory or spatial orientation at the tested doses (Fig. 3).

**Fig. 4:**
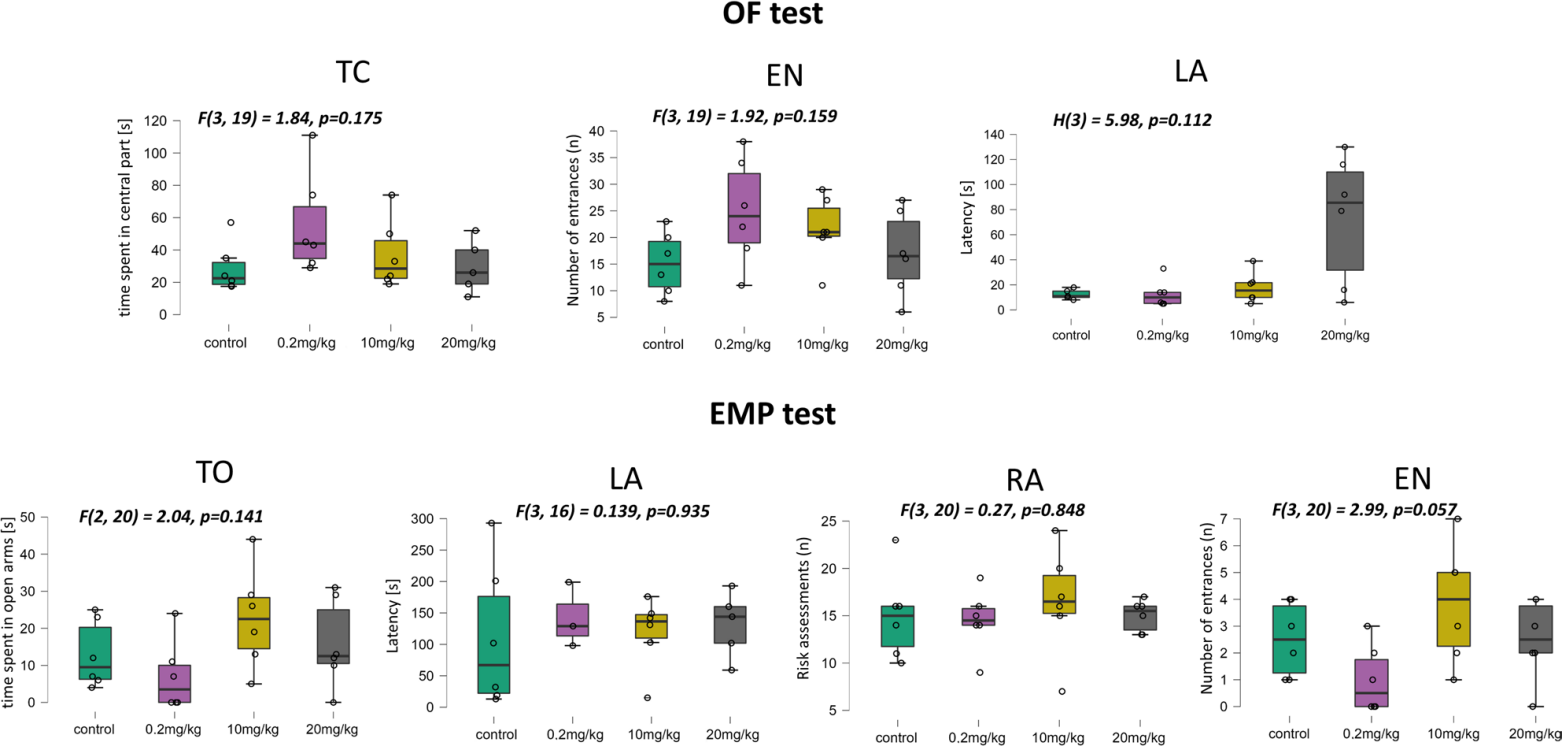
Results of open field (OF) and elevated plus maze (EPM) behavioural tests. TC - time spent in the central part; EN - number of entrances in the central part; LA -latency; TO - time spent in open arms; RA - risk assessments; EN - number of entrances in open arms; *p* – *p*-value of ANOVA (F) or Kruskal-Wallis (H) test; F(df1, df2) – ANOVA test statistics with the presented degrees of freedom; H(df1) - Kruskal-Wallis test statistics with the presented degrees of freedom

In the Open Field test and Elevated plus Maze performance, none of the analyzed parameters (OF: time spent in central, number of entrances in central part, latency) (EPM: time spent in open arms, latency, risk assessment, number of entrances in open arms) showed statistically significant differences (*p* > 0.05) between CBD-treated groups and controls, indicating no measurable effect of CBD on general locomotor activity, anxiety like behavior or exploratory behavior (Fig. 4).

### Sequencing and read statistics

Sequencing of all 48 hippocampus samples generated more than 1.4 G of PE reads, from 21.8 M to 42.0 M PE reads for individual samples. Of the reads, on average, 29.5 M per sample (98.3%) passed initial filtering. Of the filtered reads, a mean of 22.5 M reads per sample (76%) was uniquely mapped against the reference genome, and on average, 83% of them were assigned to genes specified in the applied annotation. Because of the lowered mapping rate (< 40% of uniquely mapped reads), four samples were removed from further analysis during the final RNA-Seq quality control step (Supplementary File 2).

### Alterations in hippocampus transcriptome following CBD treatment

**Fig. 5:**
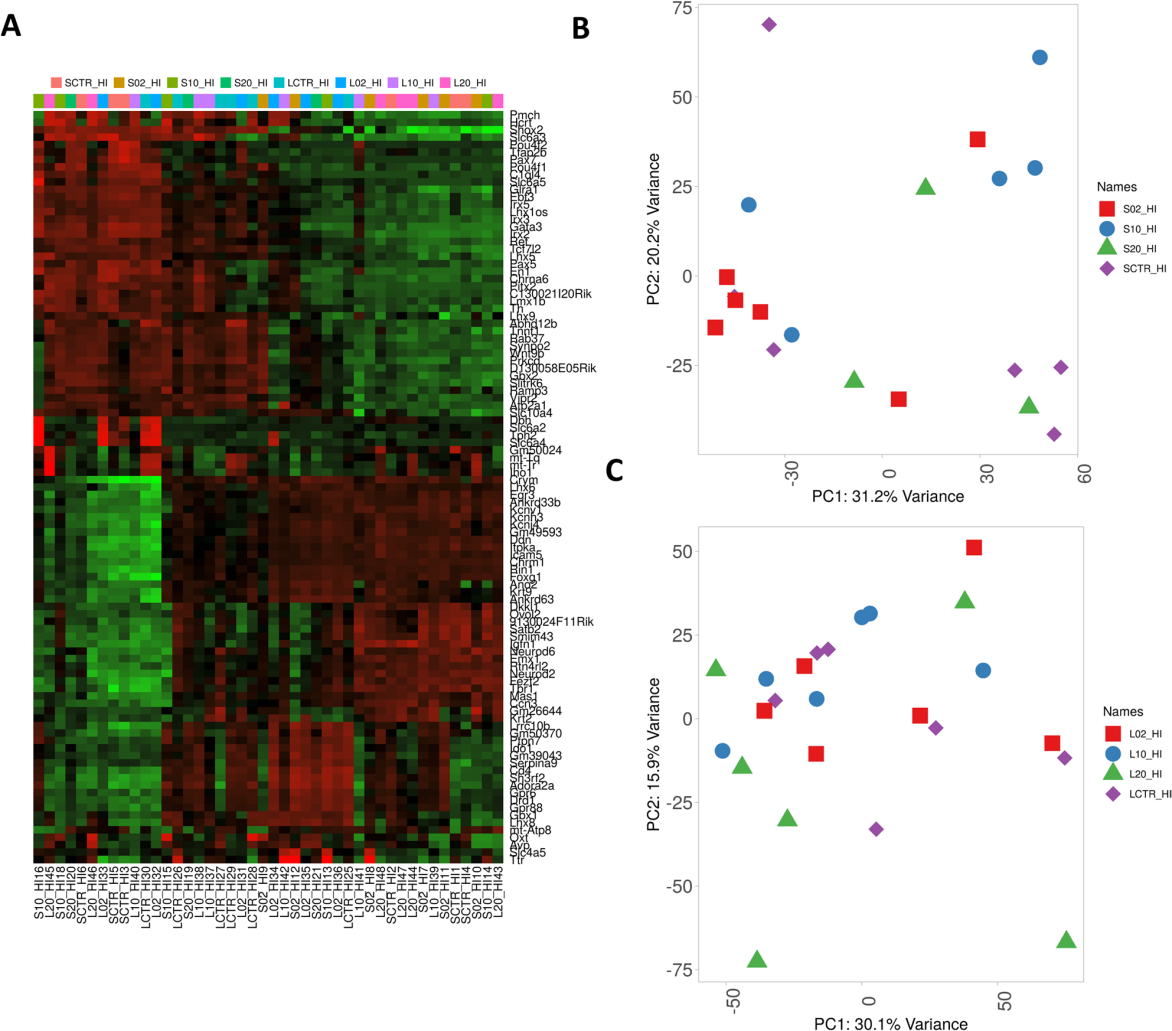
Gene expression profile differentiation for the analysed samples. **A** - heatmap with hierarchical clustering of samples and gene expression profiles. **B** - PCA for short-term (S) CBD-treated mouse hippocampus; **C** - PCA for long-term (L) treated mouse hippocampus

Hierarchical clustering using Euclidean distance of samples’ expression profiles based on 100 the most variable genes showed subdivision of samples into three major clades; however, they did not correspond to the applied CBD doses or time of its administration, suggesting that the general expression profile of the hippocampus was not severely affected by a CBD treatment (Fig. 5A). This result was confirmed by the PCA analysis performed on all genes for short-term and long-term treated mice separately. In both these results, samples were located in mixed clusters, suggesting a lack of large-scale global transcriptomic shifts associated with CBD treatment in the mouse hippocampus (Fig. 5B and C).

The differential expression analysis among the study groups showed that only very few genes were altered in the mouse hippocampus by the applied CBD treatment. In ST-treated mice, only three genes (*Polr1f*,* Tfap2b*,* Evx1os*) were downregulated by a 0.2 mg/kg b.w. CBD treatment. 10 mg/kg b.w. CBD treatment caused downregulation of *Ube2q2* and *Ipo7*, and upregulation of *Cnppd1*. No significantly altered genes were detected for 20 mg/kg b.w. treatment at an ST treatment.

**Fig. 6:**
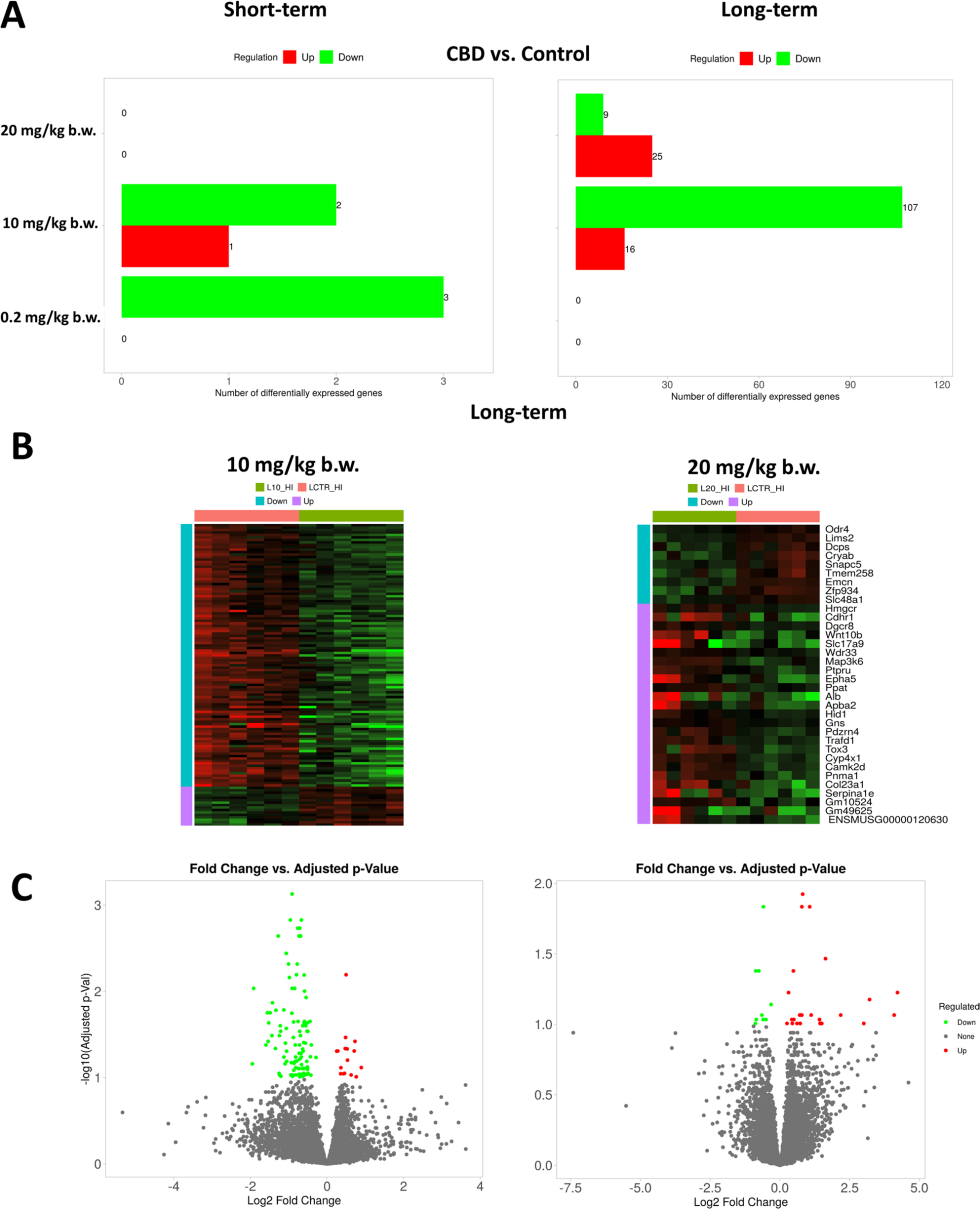
Results of differential expression analysis in the hippocampus between CBD-treated and control mice. **A** - amount of differentially expressed genes for each treatment versus control; **B** - heatmap for differentially expressed genes in long-term treatment (only doses of 10 and 20 mg/kg b.w. CBD); **C** - Volcano plot for differences in gene expression in mouse hippocampus between treated and control mice (10 and 20 mg/kg b.w. CBD)

LT CBD treatment resulted in more pronounced changes in gene expression. While the lowest applied dose did not alter the expression of any genes, 10 mg/kg b.w. affected 123 genes (107 down- and 16 up-regulated). The highest applied CBD dose, however, affected the lower number of genes (*n* = 34) and most of them were upregulated (*n* = 25) (Fig. 6, Supplementary File 3). Ten genes were common for both LT treatments (Supplementary File 3).

### Functional annotation of genes altered by CBD in mouse hippocampus

Overrepresentation tests were conducted for genes altered in LT treatment to elucidate the functional significance of transcriptional changes induced by CBD in the mouse hippocampus. No overrepresentation tests were performed for genes affected by ST treatment due to their low number and potentially unreliable results.

**Fig. 7:**
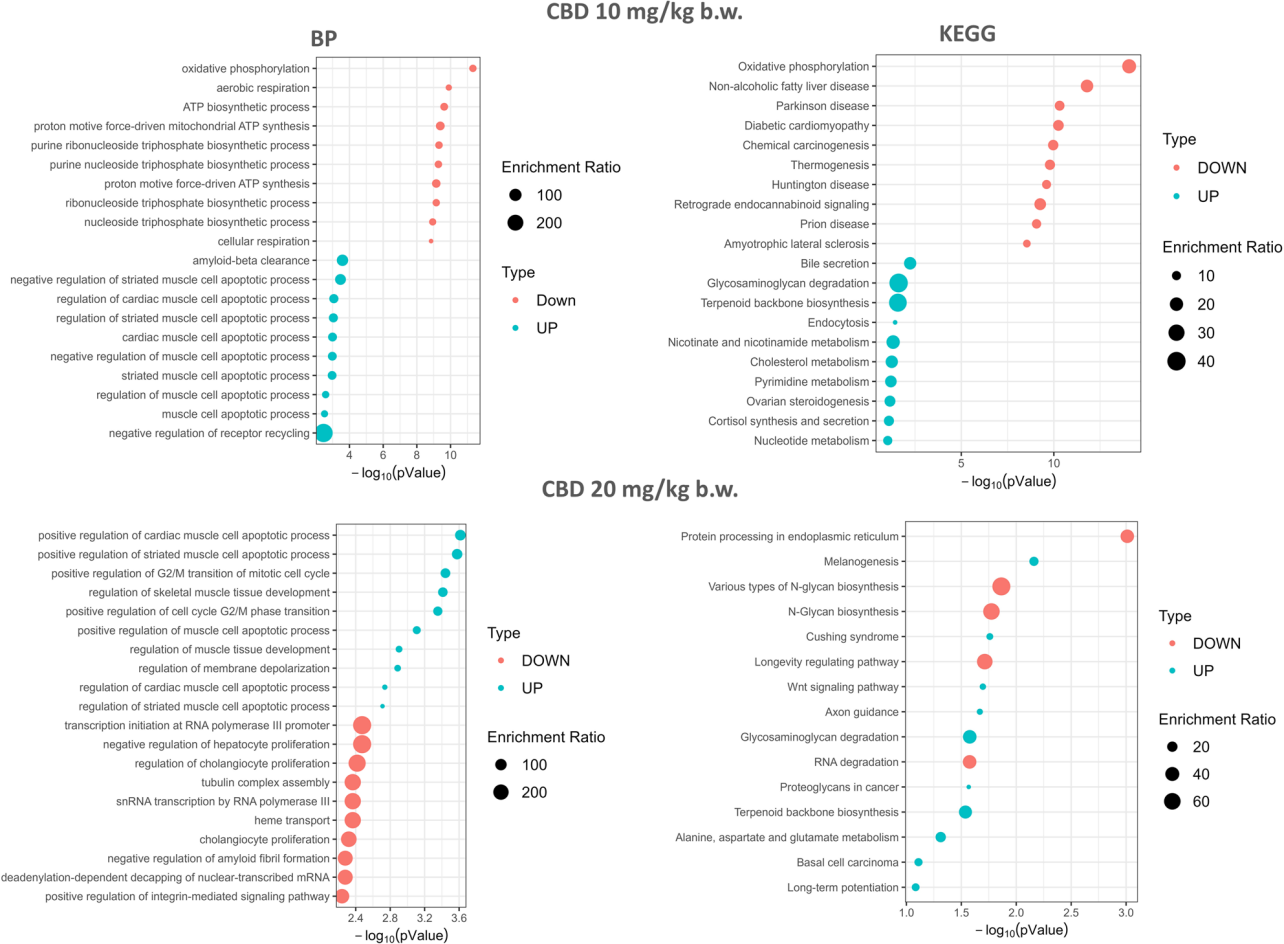
Top 10 Biological processes and KEGG pathways enriched by genes differentially expressed in the hippocampus of long-term CBD-treated vs. control mice (only doses of 10 and 20 mg/kg b.w. CBD)

The genes downregulated by a 10 mg/kg b.w. CBD dose in LT treatment significantly enriched biological processes related to oxidative phosphorylation (GO:0006119; FDR = 4.29E-08) and several other processes associated with energy metabolism and ATP synthesis. These included aerobic respiration (GO:0009060; FDR = 5.79E-07), ATP biosynthetic process (GO:0006754; FDR = 7.25E-07), mitochondrial respiratory chain complex assembly (GO:0033108; FDR = 6.64E-06), NADH dehydrogenase complex assembly (GO:0010257; FDR = 8.27E-06), and others (Supplementary File 4; Fig. 7). The upregulated genes did not significantly enrich any BP categories after correction for multiple testing. However, unadjusted p-values indicated potential involvement in processes such as amyloid-beta clearance (GO:0097242; p-value = 2.60E-04), amyloid-beta clearance via cellular catabolic processes (GO:0150094; p-value = 0.005), and several processes related to the regulation of apoptotic processes, primarily in cardiac muscle (GO:0010665, GO:0010662, GO:0010659, GO:0010656, GO:0010658, GO:0010660, GO:0010657; p-value < 0.01). Other enriched processes included the sterol metabolic process (GO:0016125; p-value < 0.005) and negative regulation of neurogenesis and nervous system development (GO:0050768, GO:0051961; p-value < 0.01) (Supplementary File 4; Fig. 7).

The genes downregulated by the 10 mg/kg b.w. CBD dose in LT treatment also significantly enriched several KEGG pathways, including oxidative phosphorylation (mmu00190; FDR = 2.98E-12), non-alcoholic fatty liver disease (mmu04932; FDR = 2.81E-10), Parkinson’s disease (mmu05012; FDR = 4.94E-09), prion disease (mmu05020; FDR = 3.32E-08), pathways of neurodegeneration (mmu05022; FDR = 1.59E-06), ribosome (mmu03010; FDR = 2.04E-05), metabolic pathways (mmu01100; FDR = 0.001), and many others. Upregulated genes enriched KEGG pathways related to bile secretion (mmu04976; FDR = 0.006), glycosaminoglycan degradation (mmu00531; FDR = 0.02), terpenoid backbone biosynthesis (mmu00900; FDR = 0.03), cholesterol metabolism (mmu04979; FDR = 0.06), and more (Supplementary File 4; Fig. 7).

The genes downregulated by a 20 mg/kg b.w. CBD dose in LT treatment did not significantly enrich any BP terms after correction for multiple testing. Nevertheless, some interesting processes with significant unadjusted p-values were observed, including tubulin complex assembly (GO:0007021; p-value = 0.004), negative regulation of amyloid fibril formation (GO:1905907; p-value = 0.005), and positive regulation of the integrin-mediated signalling pathway (GO:2001046; p-value = 0.006), among others. Similarly, the upregulated genes did not significantly enrich any processes after FDR correction. However, unadjusted p-values indicated significant associations with several processes, including striated muscle cell apoptotic processes (positive regulation) (GO:0010666, GO:0010663, GO:0010661, GO:0010665; p-value < 0.01), positive regulation of mitotic cell cycle phase transition (GO:0010971, GO:1902751, GO:0010389; p-value < 0.01), and regulation of insulin secretion in response to glucose stimulus (GO:0061178; p-value < 0.003) (Supplementary File 4; Fig. 7).

Genes affected by the 20 mg/kg b.w. CBD dose also did not significantly enrich any KEGG categories after FDR adjustment for multiple testing. However, some unadjusted p-values were significant. Among the downregulated genes, enriched pathways included protein processing in the endoplasmic reticulum (mmu04141; p-value = 9.76E-04), N-glycan biosynthesis metabolism (mmu00513, mmu00510; p-value < 0.05), and RNA degradation (mmu03018; p-value = 0.02). Meanwhile, the upregulated genes were associated with pathways related to Cushing’s syndrome (mmu04934; p-value = 0.017), Wnt signaling pathway (mmu04310; p-value = 0.02), axon guidance (mmu04360; p-value = 0.02), glycosaminoglycan degradation (mmu00531; p-value = 0.026), and others (Supplementary File 4; Fig. 7).

### qPCR validation results

The comparative analysis of RNA-Seq with qPCR yielded satisfactory results in terms of concordance; however, the strength of correlation depended on the internal control used. When using the mean expression of both control genes, the correlation coefficients were high for four of the analysed genes (in a range of 0.52 to 0.80). For one of the genes (Wrd33), it was low and reached 0.26 (Supplementary File 1).

## Discussion

This study examined the effect of intraperitoneally administered CBD at varying doses and durations on hippocampus gene expression patterns under physiological conditions. The main finding was that substantial transcriptional changes occurred just following long-term delivery of 10 mg/kg CBD, while short-term treatment did not yield significant transcriptome alterations. This selective response suggests that prolonged exposure, rather than immediate pharmacological effects, is necessary to achieve persistent molecular adaptation in the hippocampus.

The observed time and dosage specificity correspond with the intricate pharmacokinetic profile of CBD. Cannabidiol exhibits significant lipophilicity, resulting in its accumulation in peripheral organs, including adipose tissue and the liver, which causes gradual redistribution and modified steady-state exposure upon repeated treatment [29]. Simultaneously, clinical trials have revealed an inverted U-shaped dose–response curve, demonstrating that intermediate doses have maximal biological effects, whereas both lower and higher doses are less efficacious [30, 31]. Collectively, these data support the hypothesis that both cumulative exposure and dose magnitude are pivotal in determining key biological outcomes. The absence of detectable transcriptional remodeling following short-term administration is indicative of the need for protracted exposure to achieve a molecularly effective range in the brain.

A significant element of the transcriptional response to long-term 10 mg/kg CBD belonged to mitochondrial oxidative phosphorylation. Enrichment was directly detected for the oxidative phosphorylation pathway in KEGG (mmu00190) and for the associated Gene Ontology biological process (GO:0006119). Furthermore, KEGG categories identified as retrograde endocannabinoid signaling (mmu04723) and neurodegenerative pathways (mmu05022) reached statistical significance. The examination of the underlying gene sets indicated that the enrichment of these pathways was mostly influenced by a common group of mitochondrial respiratory chain genes. This encompassed several subunits of complex I from the *Ndufa*, *Ndufb*, and *Ndufs* families, in addition to specific components of cytochrome c oxidase, namely *Cox6b1* and *Cox7a2*, along with the assembly factor *Uqcc2*. These genes represent essential structural components of the oxidative phosphorylation apparatus and are simultaneously annotated across multiple KEGG pathways due to their common mitochondrial architecture. The enrichment signal thus indicates the coordinated regulation of respiratory chain modules instead of the activation of disease-specific pathways. Complex I, or NADH: ubiquinone oxidoreductase, serves as the principal entry site for electrons into the respiratory chain and is a crucial factor in the efficiency of proton pumping and ATP synthesis [32]. The correct assembly and stoichiometric balance of *Nduf* accessory subunits have significance for the stability and functional integrity of the holoenzyme [33, 34]. In neurons, nuanced alterations in complex I composition affect mitochondrial membrane potential, redox equilibrium, and the production of reactive oxygen species [35]. Due to the hippocampus’s significantly elevated metabolic requirements, even slight alterations in respiratory chain expression can result in functional metabolic adjustments. Transcriptomic evidence from alternative models further supports the susceptibility of oxidative phosphorylation pathways to stress-induced regulation. Single-nucleus sequencing in a Post-traumatic stress disorder (PTSD) mouse model demonstrated the downregulation of complex I and other oxidative phosphorylation genes in hippocampus neurons, while CBD therapy rectified this trend and enhanced the expression of respiratory chain components [36]. Despite our analysis being performed under healthy conditions rather than stress-induced pathological states, the alignment of mitochondrial respiratory genes indicates that oxidative phosphorylation may be a consistent target of CBD-mediated molecular adaptation in the hippocampus. Studies conducted in cellular models indicate that CBD may affect mitochondrial respiration and ATP-linked oxygen consumption in a concentration-dependent manner [37]. Collectively, these data establish mitochondrial bioenergetics as a pivotal element in the neuromolecular response to prolonged cannabis treatment.

In addition to oxidative phosphorylation, prolonged administration of 10 mg/kg CBD influenced genes related to processes associated with purine and nucleotide metabolism. Multiple enriched Gene Ontology terms were linked to purine ribonucleotide biosynthesis and purine nucleoside triphosphate metabolic pathways. Purine nucleotides function as substrates for ATP synthesis and are required for RNA synthesis and intracellular signaling. In hippocampal neurons, enzymes involved in the de novo purine biosynthesis pathway have been observed to concentrate near mitochondria, indicating a functional link between nucleotide synthesis and bioenergetic activity [38]. The coordinated regulation of oxidative phosphorylation and purine metabolism thus strengthens the concept of integrated metabolic adjustment rather than the activation of separate pathways. This linkage aligns with the adaptation of substrate availability, energy production, and transcriptional capability with prolonged cannabis exposure. Endocrine indicators further confirm the temporal characteristics of the reported effects.

Acute CBD delivery resulted in elevated plasma corticosterone; however, prolonged exposure caused decreased corticosterone levels at subsequent time points for both 10 and 20 mg/kg dosages. The increase in plasma corticosterone in short-term or high-dose CBD treatment was previously described in rodents [39, 40] and in humans (cortisol and corticosterone) [41]. Some studies are also available, showing decreased corticosterone levels following CBD injection in mice [42]. These data suggest that CBD effect on plasma corticosterone may be time- and dose-dependent or may depend on several other model-related factors that are difficult to grasp. The hippocampus is pivotal in the feedback regulation of the hypothalamic–pituitary–adrenal axis, and glucocorticoids are recognized for their direct impact on mitochondrial metabolism and oxidative equilibrium. Acute spikes in glucocorticoids increase metabolic demand, however extended normalization may allow for structural and functional adaptations of mitochondria. The gap between prompt endocrine activation and subsequent transcriptional changes indicates that mitochondrial gene regulation may arise following prolonged alterations in stress-axis signaling rather than from acute hormonal fluctuations.

Behavioral outcomes were limited and specific. Only one behavioral paradigm demonstrated statistically significant alterations, but spatial memory and general locomotor parameters remained predominantly unaffected. These results are in agreement with previous findings in which prolonged cannabidiol treatment did not affect memory, motor performance and anxiety in C57BL/6J mice [43]. Also, no behavioural alterations were found in normal healthy mice in other studies [44], but several pieces of evidence are present regarding the modulation of behaviour in addiction, stress or anxiety-related mouse/rat models [45–47]. The restricted behavioral phenotype found in this study aligns with the lack of acute transcriptional disruption and reinforces the notion that long-term administration of 10 mg/kg CBD results in nuanced metabolic adjustments rather than extensive neuromolecular remodeling. The integration of endocrine normalization and mitochondrial gene regulation, absent significant behavioral changes, indicates a stabilisation of the hippocampus energy state rather than explicit behavioral modulation.

The current data suggest that prolonged treatment of 10 mg/kg CBD promotes coordinated transcriptional adaptation in the hippocampus, focusing on mitochondrial oxidative phosphorylation and purine metabolism. These alterations are temporally independent of acute endocrine reactions and align with cumulative pharmacokinetic exposure and non-linear dose–response properties of CBD. It appears that sustained cannabinoid signaling engages bioenergetic recalibration within stress-sensitive hippocampal circuits, rather than triggering immediate transcriptional perturbation or neurodegenerative activation.

A limitation of the current study is that testing occurred concurrently with CBD administration, lacking a washout period. Consequently, the observed effects probably represent a mixture of acute and cumulative (subchronic) effects instead of solely chronic adaptations. Also, behavioral tests done at different times may be linked to different stages of treatment, which should be taken into account when looking at the results. Subsequent research ought to incorporate post-treatment or washout-based evaluations to more effectively delineate chronic effects. Another limitation of the present study is the relatively small sample size and the small magnitude of transcriptional changes observed following CBD treatment. Differential expression analysis revealed subsets of significantly altered genes, but the overall number of differentially expressed genes was rather low, and unsupervised analyses (PCA and hierarchical clustering) did not show clear separation between experimental groups. Thus, the effects observed should be considered as subtle modulations of the transcriptome and not as a large scale global reprogramming. In addition, the functional level interpretations are based on enrichment analysis and not on direct functional evidence. Thus, observations of coordinated bioenergetic or metabolic adaptations should be treated as hypothesis-generating rather than conclusive and need further validation using targeted functional and biochemical approaches.

As a further limitation of the study, it can also be considered the exclusive use of male mice. Given known sex-dependent differences in endocannabinoid signalling, HPA axis regulation, and behavioral responses, it cannot be excluded that females would respond differently to CBD treatment. Future studies should include both sexes to assess potential sex-specific effects.

## Conclusions

Long-term treatment of 10 mg/kg cannabidiol modulates expression of genes related to mitochondrial oxidative phosphorylation and purine metabolism in the hippocampus in a coordinated manner. These alterations presumably indicate adaptive bioenergetic recalibration associated with prolonged exposure and endocrine normalization, rather than acute pharmacological or neurotoxic consequences.

## Supplementary Information

Supplementary Material 1: Primers for qPCR and results of comparative analysis with RNA-Seq.

Supplementary Material 2: Sequencing reads statistics.

Supplementary Material 3: Results of differential expression analysis among treated and control mice.

Supplementary Material 4: Results of gene set enrichment analysis for altered genes.

## Data Availability

Raw sequencing reads were deposited in the Gene Expression Omnibus (GEO) NCBI database, under accession number GSE276463 and Sort Read Archive (SRA) database with BioProject number PRJNA1157422.
